# Improvement of Expressed Breast Milk in Mothers of Preterm Infants by Recording Breast Milk Pumping Diaries in a Neonatal Center in China

**DOI:** 10.1371/journal.pone.0144123

**Published:** 2015-12-04

**Authors:** Bin Wu, Jinxia Zheng, Ming Zhou, Xiaohong Xi, Qin Wang, Jing Hua, Xuefeng Hu, Jiang-Qin Liu

**Affiliations:** 1 Department of Neonatology, Shanghai First Maternity and Infant Hospital, Tongji University School of Medicine, Shanghai, People’s Republic of China; 2 Department of Maternal and Child Health, Shanghai First Maternity and Infant Hospital, Tongji University School of Medicine, Shanghai, People’s Republic of China; Centre Hospitalier Universitaire Vaudois, FRANCE

## Abstract

**Objectives:**

Because inadequate expression of human milk (EBM) in mothers of hospitalized infants were noticed in a neonatal center of our hospital, family education program was carried out to increase the EBM.

**Methods:**

A breast milk pumping diary was introduced to the mothers with preterm infant(s) admitted in the NICU. The ratios of EBM (days of EBM to NICU/hospitalized days), breast milk feeding (BMF) (days of infants fed with exclusive human milk/hospitalized days), mixed feeding (MF) (days of infants fed with partial breast milk and partial formula/hospitalized days), and formula feeding (FF) (days of infants fed with preterm formula/hospitalized days) were evaluated.

**Results:**

During January to April, 2014, the ratios of EBM to the NICU, BMF, MF and FF were 28.11%, 6.6%, 32.8% and 60.6%, respectively. After the introduction of breast milk pumping diary to the mothers from May 2014, the ratio of EBM to the NICU increased significantly to 53.3% (p<0.01) within the following eight months. Both the ratios of BMF and MF also rose to 23.8% and MF 55.3%, respectively. Consequently, the ratio of FF was reduced to 20.9%. Exclusive breast milk feeding also significantly reduce the duration of nil per oral (NPO) of the very low birth weight infants during hospital stay as compared to those fed with mixed feeding and formula feeding.

**Conclusion:**

The introduction of a breast milk pumping diary was associated with a significant increase in the intake of EBM of the hospitalized preterm newborns.

## Introduction

Human milk feeding not only decreases the morbidities of preterm newborns by discharge in neonatal intensive care unit, including retinopathy of prematurity (ROP) [1], infectious diseases [2] and necrotizing enterocolitis (NEC) [3], but also affects the long-term development in their later lives [4,5]. The benefits of human milk feeding have been observed in very low birth weight infants fed with their own mother’s milk [6,7], and in preterm infants fed with donor human milk [8]. Although it is possible that human milk feeding may increase the risk of low growth velocity [8] and transmission of Cytomegalovirus (CMV) to preterm infants [9], increasing awareness of human milk feeding to preterm infants has been significantly improved worldwide in recent years. The strategy of human milk feeding to preterm infants has to be developed by NICU medical staff who investigated lots of methods to promote the human milk feeding, such as antenatal lactation consult, playing music when pumping, frequent education and lactation instruction, and so on. However, the rate of exclusive human milk feeding varies largely from NICU to NICU due to how much milk the mother can pump and the process for delivering milk to NICU daily when banked human milk is not available. It has been reported that the rate of expressed breast milk (EBM) is about 15% in the NICU without human milk bank and 30% in that with donor human milk bank [10].

Delivering EBM to NICU is challenging for the parents who separated from their infants in the first several days after birth. For various reasons, mothers of premature infants face multiple challenges in establishing and maintaining an adequate supply of milk. These include delayed secretory activation, insufficient milk volume, and difficulties in milk expression due to stress or inadequate support [11]. It is very often that the mothers of preterm infants have difficulty to focus on pumping immediately after birth of the preterm baby [12]. Therefore, maintaining a high frequency of milk pumping per day could be a big impact on providing adequate milk and affecting the expression milk to NICU [13]. However, this issue usually was ignored by parents and medical staff [14]. Here we demonstrated a simple dairy recorded by mother can improve the rate of human milk feeding in a neonatal center without human milk bank in China.

## Methods

This is a retrospective study. The department of neonatology of our hospital is 20 NICU beds and 60 nursery beds unit. Data of mothers and newborns were collected by medical chart review. Only infants admitted to the NICU between January 1^st^, 2014 and December 31^st^, 2014 were included in the study. Infants transferred to other children’s hospitals due to cardiac, gastrointestinal, or other abnormalities within first week of life were excluded. This study was approved by Ethics Committee of Tongji University School of Medicine. All the patient records/information was anonymized and de-identified prior to analysis. The approval letter was uploaded in the attached files.

When a preterm infant was admitted into the unit, the parents were informed about the lactation by nurses. This included the importance of breastfeeding, methods of pumping, storage and delivery of the milk. Once the feeding of the preterm infant was initiated, the parents were notified to deliver the expressed breast milk to the ward. Providing that the cold chain (the EBM be stored and delivered at 4–8°C) was maintained, the EBM was taken into the ward and marked with mother’s name and pumping date. The milk was then stored in a refrigerator at 4–8°C. Only human milk from preterm infant’s own mother pumped within 24 hours was used to feed the infant without pasteurization. EBM could be sent twice daily, 9 am and 4 pm. Parents were encouraged to deliver EBM at least once daily.

### Breast milk pumping diary

**Fig 1:**
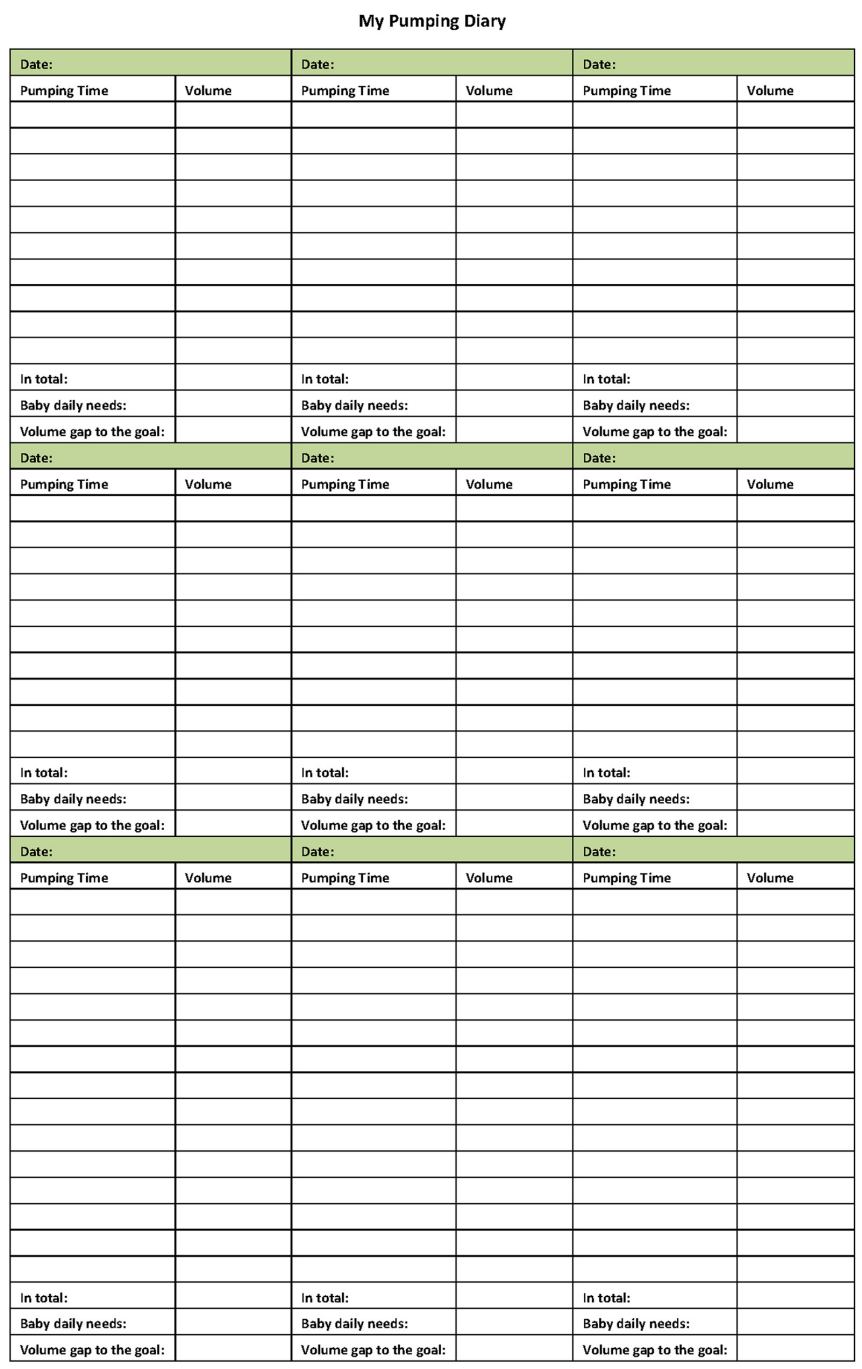
Front page of the breast milk pumping diary.

**Fig 2:**
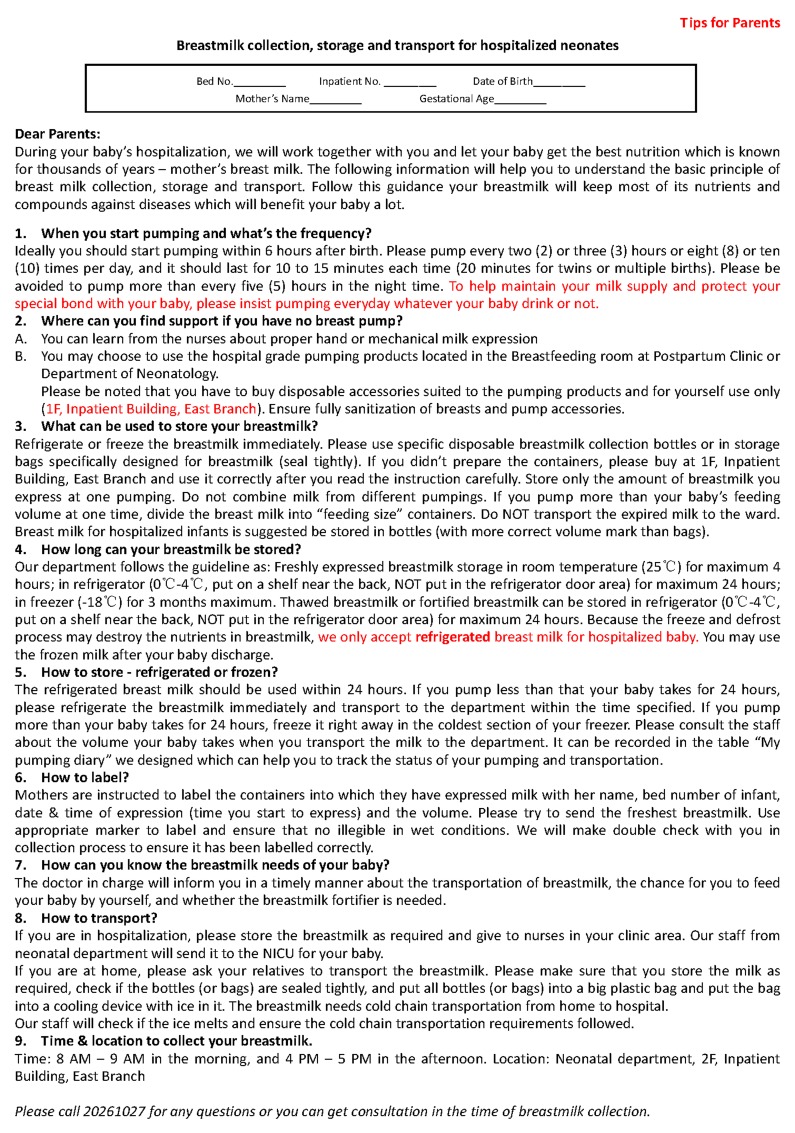
Back page of the breast milk pumping diary.

In April of 2014, the rate of breast milk feeding was not increasing and inadequate delivery of EBM to the NICU was noticed. A quality control group was established in order to improve the breast milk feeding in the NICU. In addition to strengthening the education of lactation, a breast milk pumping diary was designed to encourage the mother of a preterm infant to pump frequently. This diary contained all the important information of pumping, including the date, time and milk volume of each pumping (Figs 1 and 2, front and back pages). The total amount of milk pumped within that day was summarized at the bottom of the recorded diary. The total milk feeding volume of the baby within the same day was also recorded in the diary. Accordingly, the difference of milk amount between pumping and feeding was then calculated.

The breast milk pumping diary was provided to parents on the first day that the infant was admitted to the NICU on the first day. As described above, breastfeeding education was provided to the parents by a nurse. Pumping of breast milk more than 8 times per day was emphasized to every mother. The mother was asked to record the detail information in the diary: the date, the time and volume of every pumping, daily total volume. The diary would be reviewed by medical staff every time when the EBM was delivered to NICU.

All the preterm infants were tended with a standardized medical care and were fed every 2–3 hours, 8–12 times a day (24 hours). Each infant has a daily feeding record to show type of feeds for each feeding. BMF refers to a preterm infant took all his/her mother’s milk in a day, with or without human milk fortifier.MF refers to a preterm infant took at least one feeds of human milk or formula feeding within one day. FF refers to all the feeds were formula in a day on the record. The ratios of BMF, MF and FF were calculated as follow:

The ratio of BMF=days of infants fed with BMFhospitalized days

The ratio of MF=days of infants fed with MFhospitalized days

The ratio of FF=days of infants fed with FFhospitalized days

The ratio of EBM=days of EBM to NICUtotal hospitalized days

Breast milk delivered to the NICU at least once a day, this day was counted as EBM to NICU day.

### Breast milk feeding for very low birth weight infants (VLBWI)

Apart from the aforementioned record of EBM, the charts of VLBWIs were reviewed and the methods of feeding were analyzed. The breast milk feeding (BMF) refers to that the infant was only fed by breast milk from second week of life to discharge. Mixed feeding (MF) refers to that the infant was fed by at least one breast milk and one formula during the hospitalization. Formula feeding (FF) refers to that the infant was fed by preterm formula without any breast milk during the hospitalization. The clinical data was collected and the outcomes by discharge were also analyzed.

### Statistical analyses

SPSS version 21.0 was used for statistical analyses. Descriptive statistics were used to describe mother and infant characteristics. The normally distributed results are reported with mean and standard deviation (SD); the remaining results are reported with median, interquartile range (IQR) or percentages. One-way ANOVA was used to determine statistically significant differences in normally distributed scale data between groups. Chi-Square test was used to determine statistically significant differences for nominal data.

## Results

Overall, 12,718 infants were delivered in 2014 in this hospital and 1909 infants were admitted into the Department of Neonatology, including 812 preterm infants.

### Effects of pumping diary on breast milk feeding to preterm infants

Between Jan and Apr in 2014 (before the pumping diary), the average ratio of EBM was 28.1% (Table 1). As shown in Table 1, it significantly increased to 53.3% after the pumping diary introduced (between May and Dec in 2014, p<0.01).

**Table 1:** Monthly rate of expression of human milk and constituent ratio of feeds of preterm infants in 2014. Abbreviation: EHM, expressed human milk; BMF, breast milk feeding; MF, mixed feeding; FF, formula feeding

Month	Hospital days	EHM		BMF		MF		FF	
		days	%	days	%	days	%	days	%
**Jan**	727	181	24.9	36	4.9	199	27.4	492	67.8
**Far**	878	228	26.0	38	4.3	290	33.0	550	64.3
**Mar**	1152	357	31.0	92	8.0	377	32.1	683	59.0
**Apr**	1124	325	28.9	89	7.9	408	36.3	627	55.8
**May**	1135	526	46.3	137	12.1	562	49.5	436	38.4
**Jun**	1391	615	44.2	154	11.1	763	54.9	474	34.1
**Jul**	1393	667	47.9	169	12.1	716	51.4	508	36.6
**Aug**	1486	757	50.9	238	16.2	927	62.4	321	21.4
**Sep**	1360	761	56.0	286	21.0	784	57.7	290	21.4
**Oct**	1655	952	57.5	608	36.7	951	57.5	96	5.8
**Nov**	1571	910	57.9	489	31.1	913	58.1	169	10.8
**Dec**	1564	972	62.1	672	43.0	770	49.2	122	7.8

**Fig 3:**
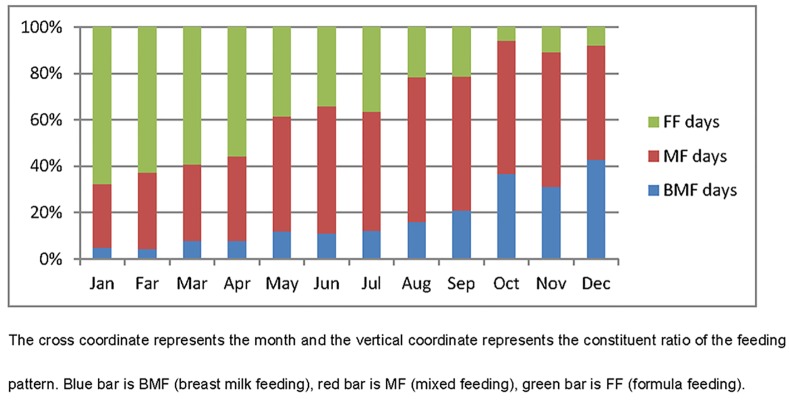
Monthly constituent ratio of feeds of preterm infants in 2014 (blue bar is BMF, red bar is MF, and green bar is FF).

According to the number of times of breast milk or formula feeding, the preterm infants were divided into three groups: breast milk feeding (BMF), mixed feeding(MF), and formula feeding (FF). Between January and April (before the introduction of pumping diary), the constitution ratios of BMF, MF and FF were 6.6%, 32.8% and 60.6%, respectively (Table 1 and Fig 3). After the introduction of breast diary, both ratios of BMF (23.8%) and MF (55.3%) were increased. Consequently, the ratio of FF (20.9%) decreased significantly (Table 1 and Fig 3).

### Effects of breast milk feeding on the outcomes by discharge of the very low birth weight infants

There were 141 very low birth weight infants delivered in this hospital, 29 infants were transferred to other children’s hospital for the reasons mentioned above. Of the 112 VLBWI who stayed in our department, 93 survived and 19 died.The overall survival rate was 83.0%. The infants were divided into 3 groups according to the methods of feedings: BMF (14 infants), MF (45 infants) and FF (34 infants). The demographic data of these infants was presented in Table 2. The exclusive breastfeeding rate of these VLBWI was 15.1%.

**Table 2:** Demography of survives of very low birth weight infants.

Factors	FF (n = 34)	MF (n = 45)	BMF (n = 14)	F/H/χ^2^	P
Gestational Age (weeks)	30.40±2.26	30.35±2.26	29.86±3.06	0.28	0.76
BirthWeight (grams)	1200±176	1235±197	1266±166	0.70	0.50
Gender (male)	41.2%(14)	48.9%(22)	50.0%(7)	0.56	0.76
twin	44.1%(15)	33.3%(15)	35.7%(5)	0.99	0.61
Apgar at 1 min	8[6, 9]	8[6, 9]	8[7, 9]	0.20	0.90
Apgar at 5 min	9[8, 10]	9[8, 10]	9[9, 9]	0.16	0.92
Delivery by cesarean section	58.8%(20)	55.6%(25)	57.1%(8)	0.08	0.96
Temprature at admission	35.8±0.5	36.1±0.5	35.9±0.6	2.31	0.11

Thirty-seven VLBWI were admitted within January 1^st^ to April 30^th^, 2014, and 56 were admitted within May 1^st^ to December 31^st^, 2014. Before the pumping diary was used, the ratios of BMF, MF and FF of VLBWI were 2.7% (1/37), 35.1% (13/37) and 62.2% (23/37), respectively. After the May of 2014, the ratios of BMF, MF and FF were 23.2% (13/56), 57.2% (32/56) and 19.6% (11/56), respectively (p<0.001).

As shown in Table 3, the days on NPO (nil per oral) of BMF infants were significantly shorter than the other two groups (P<0.05). Even though there were not statistically different, the BMF group had the shorter PN, CVC and hospitalized days, lower NEC, ROP, brain injury, infectious disease and medical cost by discharge as compared to the other two groups (Table 3).

**Table 3:** Clinical factors and outcomes by discharge of VLBWI. Abbreviation: BMF, breast milk feeding; MF, mixed feeding; FF, formula feeding; PN, parenteral nutrition; NPO, nil per oral; DW, discharge weight; CVC, central venous catheter; MV, mechanical ventilation; BPD, bronchopulmonary dysplasia; ROP, retinopathy of prematurity; NEC, necrotizing colitis. Infectious diseases were diagnosed by positive blood culture or clinical symptoms.

Factors	FF (n = 34)	MF (n = 45)	BMF (n = 14)	F/H/χ^2^	P
**PN(days)**	20 [12, 37]	18 [12, 30]	15 [13, 23]	1.02	0.60
**NPO(days)**	**3 [1, 9]**	**2 [1, 3]**	**1 [1,2]**	**8.03**	**0.02**
**DW(grams)**	2302±291	2254±225	2431±415	2.07	0.13
**CVC(days)**	15.5 [3, 33]	16 [10.5, 27]	10 [7, 16]	1.90	0.39
**MV scoring**	13.5 [1, 43.5]	23.5 [7.5, 40.1]	16.8 [6, 42]	0.61	0.74
**Transfusion**	0 [0, 2]	0 [0, 2]	0 [0, 2]	0.41	0.82
**Hospital stay (days)**	59.7±22.1	60.8±19.9	58.8±18.3	0.06	0.94
**Cost (thousands RMB)**	43.3±30.5	43.5±24.4	39.5±19.4	0.13	0.88
**BPD**	32.4%(11/34)	44.4%(20/45)	42.8%(6/14)	1.67	0.79
**ROP**	23.5%(8)	15.6%(7)	7.1%(1)	2.04	0.36
**Brain injury**	17.6%(6)	20%(9)	14.2%(2)	0.18	0.92
**NEC**	1	2	0	0.69	0.71
**Infectious diseases**	38.2%(13)	40%(18)	28.6%(4)	0.60	0.74

## Discussion

Using breast milk pumping diary to the mother of a preterm infant after birth significantly improved the delivery of expressed breast milk to the NICU and the rate of exclusive breast milk feeding of very low birth weight infants.

A preliminary investigation on April of 2014 in our unit was conducted by the quality control group of medical staff to identify the reasons of insufficient breast feeding of the preterm infants. We found that the major reason for parents not delivering milk to our NICU was insufficient milk production and low frequency (less than 6 times per day) of pumping [15]. To overcome this problem, a breast milk pumping diary was designed to improve the expression of breast milk. After the introduction of breast milk pumping diary to the mothers in May 2014, the ratio of EBM to the NICU increased significantly. As mothers were encouraged to pump a minimum of 8 times per day and potentially up to 12 times per day during the first several weeks when breast milk supply is being established [16], we believed that the pumping diary increased the pumping frequency of the mothers to ensure enough milk supply [13]. Frequent pumping also improves the degree of milk removal of the breasts. The mothers recorded the milk volume of every pumping, which may encourage them to emptying the breast as much as possible to increase the pumping volume [17] as well as to maintain the milk supply [13].

The first 2 weeks are a critical period for initiation and maintenance of lactation in this population. However, this issue has been overshadowed by the priorities of NICU care by both parents and clinicians [18]. Because preterm infants often do not receive full enteral feedings for the first 2 weeks, mothers may be falsely reassured that they have expressed “enough” milk during that period. Eventually, this may lead to an inadequate supply. Pumping diary can help mothers and nurses to discover the problem and take timely measures. Additionally, recording the diary can help mothers to see their efforts and achievements, resulting in pumping milk more actively. It has been reported that the provision of milk is a unique maternal activity that provides mothers with a sense of purpose and worth to make a strong bond with their infants [19,20]. This also relieves the guilt regarding the separation with their preterm infants and reinforces their role as a“good mother.” [21,22]. Recording the diary certainly strengthens this process. From the diary mothers could see what they had done for their infants, this helps mothers concentrate more on pumping. A mother once described her feeling of pumping and delivering milk like this “Even if I can’t be there for him every single day, I am pumping still. When I am pumping and recording the diary, I feel I am doing something for him. I just don’t want anything else to get in the way of pumping.”

The increase of the ratio of EBM also caused a change in the feeding patterns of preterm infants in NICU. After the introduction of pumping diary, all the breast milk related feeding patterns constituent ratios showed an upward trend, whereas the proportion of pure formula feeding correspondingly reduced. The proportion of BMF and MF increased monthly. This result suggested that pumping diary could help mothers decide to start pumping and persist in pumping during their babies stay in hospital. It also confirmed our preliminary investigation that the reason for not delivering milk was due to insufficient milk production rather than reluctance for milk delivery.

The clinical outcomes of the VLBWI in our NICU in 2014 were analyzed by feeding pattern. The PN days, NPO days, CVC days, incidence of NEC, hospitalized days and cost of the babies in EBMF group were comparatively shorter and lower than those of the babies in PFF and PBMF groups. These results were consistent with other studies [2,3] that breast milk feeding improves the outcomes of VLBW infants. Therefore, the promotion of breastfeeding in NICU is very important.

Although the milk volume of the daily pumping of each mother of a preterm infant was not recorded and the improvement of the volume in each month was not available due to the diary was noted and kept by the parents, it has been demonstrated that more parents can supply sufficient amount of milk to the NICU after the diary was introduced. Owing to the small sample size of very low birth weight infant, the outcomes such as ROP and NEC of different feeding pattern was not statistically significant. Additional data collections are required to demonstrate the improvement of the outcome of VLBWI by feeding the infants with their mother’s milk.

In summary, implementing a breast milk pumping diary can increase the ratio of EBM feeding of preterm infants in the NICU and improve the breast milk feeding of the hospitalized preterm newborns.

## Supporting Information

S1 Checklist PLOS ONE Clinical Studies Checklist. (DOCX)

S2 Checklist STROBE Statement—checklist of items that should be included in reports of observational studies. (DOC)
